# Patients’ perspectives on cancer care disparities in Central and Eastern European countries: experiencing taboos, misinformation and barriers in the healthcare system

**DOI:** 10.3389/fonc.2024.1420178

**Published:** 2024-08-09

**Authors:** Veronica Coppini, Giulia Ferraris, Maria Vittoria Ferrari, Margherita Dahò, Iva Kirac, Ira Renko, Dario Monzani, Roberto Grasso, Gabriella Pravettoni

**Affiliations:** ^1^ Applied Research Division for Cognitive and Psychological Science, European Institute of Oncology IRCCS (IEO), Milan, Italy; ^2^ Department of Psychology, Educational Science and Human Movement (SPPEFF), University of Palermo, Palermo, Italy; ^3^ Genetic Counseling Unit, University Hospital for Tumors, Sestre Milosrdnice University Hospital Center, Zagreb, Croatia; ^4^ Department of Oncology and Hemato-Oncology, University of Milan, Milan, Italy

**Keywords:** cancer disparities, cancer divide, cancer inequalities, healthcare system corruption, cancer taboo, psycho-oncology, Central-Eastern Europe, digital divide

## Abstract

**Introduction:**

Despite the advancements in oncological medicine and research, cancer remains the second leading cause of death in Europe with Central and Eastern European countries, such as Slovakia and Croatia, showing the highest mortality rates and disparities in access to appropriate and comprehensive cancer care. Therefore, the primary aim of the current study is to investigate cancer patients’ perspectives and experiences to understand the possible underlying reasons for cancer disparities.

**Methods:**

Croatian cancer patients (n=15) and Slovak patients (n=11) were recruited through social media platforms, patients’ organisations, and hospital websites and offered participation in online focus group discussions on perceived disparities, barriers or malfunctioning during and after their cancer journey. Transcripts of video and audio recordings of the interviews were translated and analysed using Thematic analysis.

**Results:**

Six Croatian and five Slovak themes emerged from the focus group discussions highlighting encountered barriers and perceived disparities, as well as suggestions or unmet needs. Most of the themes are common to both groups, such as the lack of information and use of the internet, and the taboos regarding cancer or psycho-oncological support. However, some themes are specific to each group, for instance, Slovak cancer patients remarked the fact that they do not mind travelling to get treatment as long as they can be treated in the west of Slovakia, while Croatian patients highlighted the need for more information after the illness and the socioeconomic impact deriving from a cancer diagnosis.

**Discussion:**

Urgent intervention is needed in addressing disparities in Central and Eastern Europe. Present results could inform dedicated guidelines or better resource allocation strategies to reduce disparities in cancer care and promote inclusive healthcare.

## 1 Introduction

Despite the continuous progress in oncological medicine and research, and regardless the notable decrease in mortality rates in cancer diseases, as well as increased attention towards socio-economic disparities and discriminations, cancer still remains the second leading cause of death in many countries (1). The World Health Organization (WHO), recently released the latest estimates of the global cancer burden ahead of World Cancer Day through the International Agency for Research on Cancer. These estimates, based on data from 2022, call attention to the growing challenge of cancer worldwide, particularly affecting underprivileged populations such as Central and Eastern European (CEE) countries. In addition, the WHO published findings from a survey conducted across 115 countries, revealing that the majority of nations does not adequately finance comprehensive cancer care and palliative care services as part of universal health coverage (UHC). The global survey on UHC and cancer highlighted significant disparities in cancer care provision, with only a minority of countries (39%) covering basic cancer management and palliative care services within their health benefit packages. Looking ahead to 2050, the global cancer burden is projected to increase substantially, with over 35 million new cases predicted, representing a 77% rise from 2022 estimates. This increase is expected to impact low and medium Human Development Index countries disproportionately, highlighting the urgent need for global efforts to address cancer disparities (2, 3). Shifting the focus to Europe, a persistent excess of cancer mortality in Central-Eastern compared to Western Europe, with the gap widening over time, is reported by Santucci and colleagues (4). While Western Europe saw significant declines in cancer mortality and averted 3.9 million deaths between 1990 and 2016, Central-Eastern Europe witnessed minimal progress. In 2016, over 55.000 cancer deaths in CEE countries could have been prevented by narrowing the mortality gap. Factors contributing to this discrepancy include differences in lifestyle behaviours, such as smoking and alcohol consumption, and variations in cancer diagnosis and management practices between the two regions (4). In CEE countries such as Poland and Romania, early detection programs have shown limited effectiveness, with some regions lacking the implementation of newer programs. Additionally, CEE countries face longer average diagnosis times for breast cancer, with Poland’s diagnosis period lasting up to 9.5 weeks and even reaching 38 weeks in some cases. Limited availability of advanced cancer treatment medications further hampers equal access to care in Central-Eastern Europe (5). Further examples could be Slovakia and Croatia. The cancer burden in Slovakia is an additional challenge to an already disrupted healthcare system, struggling with high mortality rates from preventable causes, including cancer. Indeed, despite Slovakia initiating its first National Oncology Program in 2018, supported by the establishment of the National Oncology Institute and the introduction of new screening protocols for mammography and colorectal cancer in January 2019, the situation remains particularly acute (6). Over 29.000 new cancer cases are projected for 2022, exceeding EU averages for men and women. Slovakia’s cancer mortality rate is among the EU’s highest, with over 13.000 deaths recorded in 2021 (7). Similarly, in Croatia, the projected number of new cancer cases in 2022 stands at over 27.000, also exceeding EU averages and placing its cancer mortality rates at the highest in Europe as well, following Hungary, recording also over 13.000 deaths in 2021 alone (8). Both countries exhibit lower five-year survival rates for cancer patients compared to the EU average, and access to treatment is limited, particularly due to geographical distance (9). Moreover, CEE countries report higher rates of risk factors such as smoking and obesity, along with poor responses to cancer screening programs. Despite the shared challenges, both Croatia and Slovakia have recognised the urgency of addressing cancer within their broader healthcare contexts. Such awareness has led to the adoption of respective national plans aimed at breaking the existing barriers in CEE countries’ healthcare systems and addressing the numerous disparities in access to cancer care (10).

While several efforts, aimed at mitigating prevailing barriers and inequities concerning access to comprehensive cancer care throughout European nations, are being carried out, a notable knowledge gap on underlying reasons behind such disparities persists particularly within Central-Eastern Europe. Therefore, the main objective of the current study is to explore the complexity of cancer-related disparities and inequalities in Slovakia and Croatia, with a specific focus on the experiences and perspectives of cancer patients regarding any barrier or difficulty encountered throughout their cancer journey. Through focus group discussions with cancer patients, the present study proposes to provide a nuanced understanding of the multifaceted dynamics shaping the landscape of cancer care and patient experiences from the Slovak and Croatian patients’ perspectives.

## 2 Materials and methods

### 2.1 Participants

Participants were recruited between May 2023 and September 2023 as part of the BEACON Cancer Care project (BEACON). Several social media platforms (Twitter, Facebook, Instagram), hospital websites and cancer patients’ organisations were consulted for the recruitment of the Slovak participants while participants from Croatia were recruited in hospitals and oncology centres. All Croatian participants, as well as 4 participants from Slovakia, were treated within public healthcare system capacities, while the remaining 7 Slovak participants were treated in a Private centre. The current study included patients 18 years of age or older, with a cancer diagnosis present or previous, caregivers of a cancer patient unable to participate, and patients residing in Croatia and Slovakia. Moreover, participants were required to speak Croatian or Slovak fluently and have technological devices with efficient internet connection that would enable participation in online focus group discussions. Lastly, cancer survivors were also included as their participation could have added richness and perspective to the results. Exclusion criteria were restricted to individuals not residing in Croatia or Slovakia, younger than 18 years of age, without internet access and those who had not received a cancer diagnosis themselves or were not caregivers to cancer patients.

### 2.2 Procedure

For the conduction of the current focus group study, the authors followed the guidelines of the Standards for reporting qualitative research [SRQR (11); see **Supplementary Material 1**]. This study was conducted within the wider structure of the BEACON project. Details of the whole BEACON study, including objectives and methodologies, are extensively described in the protocol study (12), while information from Italian focus groups with cancer patients and healthcare providers is reported in a previous focus group study within the BEACON project (13). The present study proposes to explore qualitative perspectives and insights of Croatian and Slovak cancer patients with a specific focus on their perception and their experiences within the whole cancer journey. Before the organisation and planning of the focus group discussions, ethical approval for this research was granted by the Bioethics Committee of the University of Palermo and participants provided informed consent electronically. To facilitate online discussions, participants were organised into 5 separate sessions for Croatian patients and 3 separate sessions for Slovak patients as data saturation was achieved and no new information or themes emerged. Data saturation was determined by evaluating information redundancy alongside the decreasing unfolding of new themes in the focus group discussions (14). The Croatian focus group sessions were moderated by two trained psychologists from the Klinicki bolnicki centar sestre Milosrdnice ustanova (SMUHC; Croatia) listed as authors (IK and IR), and were held in Croatian, while the Slovak focus groups, held in Slovak language, were mediated by an expert psychologist (VC) from the European Institute of Oncology of Milan who is also author of the current study. During the online discussions, the psychologists outlined the organisation of the focus group discussion, providing more detailed information about the BEACON’s wider objectives. The moderators aimed at facilitating the group dynamics to ensure that all participants would share their stories and insights freely and without any fear of judgement or repercussion from the other participants or the moderators. The entire procedure adhered meticulously to the ethical and methodological standards established in the BEACON study protocol, guaranteeing consistency and agreement with the general objectives of the project (12). A conceptual map of the recruitment flowchart is displayed in Flowchart 1. See **Supplementary Material 2**.

### 2.3 Data analysis

The analytical approach utilised in this study aligns with the qualitative thematic analysis framework, described by Clarke and Braun (15). Specifically, they suggest the following six steps: 1) Familiarisation of data; 2) Generation of codes; 3) Combining codes into themes; 4) Reviewing themes; 5) Determine the significance of themes; 6) Reporting of findings. Thus, two researchers (VC, GF) independently conducted data analysis to assure robustness and exhaustive results. Employing a bottom-up qualitative thematic analysis approach, allowed the authors to focus on letting themes to emerge organically from the data, rather than imposing a predetermined coding framework. This methodological choice facilitated a thorough exploration of participants’ experiences and perspectives. The Socioeconomic Impact (SEI) Framework (16) was considered for the contextualisation of the emerged socioeconomic aspects. Initially, following transcription, translation, and anonymisation procedures of the audio and video recordings from the focus group discussions, both authors (VC and GF) extensively familiarised with the content through multiple readings. Further, an initial coding of the data was carried out, with codes used to identify segments within the textual reports based on their semantic content, enabling systematic data organisation. In the following phase of analysis, codes were clustered into sub-themes and potential main themes focusing on spotting any disparity, difficulty, preference, or unmet need of the participants. During and after analysis, any coding discrepancies and theme identifications were resolved through discussion to ensure inter-rater reliability and analytical consistency. With the subsequent phase, emerging themes underwent further refinement and review to ensure coherence and relevance to the research question, aiming to accurately reflect the data. Lastly, themes were labelled and accompanied by explicit definitions for improved clarity and transparency in the analysis process.

To enhance the credibility and robustness of the resulting themes, two authors (MVF and DM), not involved in the initial analysis phases, carefully inspected the entire process and the identified themes, providing external validation as suggested by Clarke and Braun (15).

In this study, the focus group interview schemes included a range of open-ended questions tailored to explore various aspects pertinent to cancer patients’ experiences, including difficulties in accessing care and information, potential delays and reasons for delays in diagnosis or treatment decisions, decision-making processes, use of internet, unmet needs, and challenges in accessing psychological support. The complete interview scheme can be found in the study protocol paper (12).

## 3 Results

### 3.1 Patients’ characteristics

Five focus group discussions were held with 15 Croatian cancer patients and three focus group discussions with 11 Slovak cancer patients. The mean age of the Croatian patients was 56.9 (SD= ± 6.2) while Slovak patients’ mean age was lower (M=48.8; SD= ± 4.8). In both samples, a majority of female patients emerged (60% = Croatian female patients; 81.8% = Slovak female patients). The majority of the participants in Croatia (26.7%) had a diagnosis of breast cancer followed by colorectal (20%) and prostate cancer (20%). In Slovakia, the majority had Hodgkin’s lymphoma (63.7%) followed by breast cancer (18.2%). Lastly, half of the Croatian patients resided in cities or central areas (53.3%), the rest in suburban areas (33.3%) and a small number of patients were living in rural areas (13.3%). While the majority of Slovak patients resided in rural areas (45.5%) followed by patients living in central areas (36.4%) and in suburban areas (18.2%). Further information regarding the patient’s sociodemographic and health status is presented in **Tables 1**, **2**.

**Table 1:** Patients' sociodemographic characteristics.

Sociodemographic Characteristics	Croatian Patients n = 15		Slovak Patients n = 11	
	*n*	%	*n*	%
Gender				
Female	9	60	9	81.8
Male	6	40	2	18.2
Marital status				
Single	2	13.3	2	18.2
Married	10	66.7	9	81.8
Divorced	3	20	0	0
Highest educational level				
High school	6	40	6	54.5
University or postgraduate degree	9	60	5	45.5
Employment				
Unemployed	6	40	4	36.4
Employed full-time	7	46.7	6	54.5
Employed part-time	2	13.3	1	9.1
Ruralness of residence				
City/Central area	8	53.3	4	36.4
Suburban area	5	33.3	2	18.2
Rural area	2	13.3	5	45.5
Geographical area				
North	15	100	0	0
West	0	0	6	54.5
North-Central	0	0	3	27.3
South-Central	0	0	2	18.2

**Table 2:** Patients’ health status.

Health Status	Croatian Patients		Slovak Patients	
	*n*	%	*n*	%
Latest diagnosis				
Colorectal	3	20	0	0
Breast	4	26.7	2	18.2
Prostate	3	20	1	9.1
Cervical	1	6.7	0	0
Liver	1	6.7	0	0
Thyroid	1	6.7	0	0
Chondrosarcoma	1	6.7	0	0
Myelodysplasia	1	6.7	0	0
Hodgkin’s lymphoma	0	0	7	63.6
Mediastinal lymphoma	0	0	1	91
Time since diagnosis				
< 1 year	0	0	1	9.1
1-3 years	4	26.7	6	54.5
3-5 years	3	20	1	9.1
> 5 years	3	20	3	27.3
N/A	5	33.3	0	0
Previous diagnosis*				
No	15	100	10	90.1
Ewing’s sarcoma	0	0	1	9.1
Comorbid health diseases				
No	0	0	0	0
Chronic disease	1	6.7	1	9.1
Mental health problems	0	0	2	18.2
Physical impairment or disability	7	46.7	3	27.3
None of the above	7	46.7	5	45.5
Care and support				
No	7	46.7	6	54.5
Spouse/Partner	7	46.7	4	36.4
Friends/Family	1	6.7	1	9.1

*The Previous Diagnosis section refers to an eventual pre-existing diagnosis of cancer, different from the diagnosis at the moment of data collection.

### 3.2 Themes of focus group discussions

During the focus group discussions, all the participants expressed their opinions, experiences and feelings regarding their cancer journey in a semi-guided context. In the following paragraphs, the themes that emerged from the discussions held in the two countries will be described in detail as well as the sub-themes and some example quotes. All the themes and sub-themes will be displayed in **Table 3**.

**Table 3:** Themes and Sub-themes from Croatian and Slovak Patients.

Croatia	Slovakia
**1. The emotional impact of a cancer diagnosis and coping strategies** • The emotional burden of cancer • Lack of empathy from healthcare providers • Unequal access to psycho-oncological support • Social support, faith and religion	**1. The cancer taboo** • Extensive taboo associated with cancer • Absence of online Slovak communities or Facebook groups
**2. Socioeconomic impact** • Impossibility of getting part-time contracts or sick-leave benefits • Loss of income because of the total inability to work • Lack of awareness about financial support and reimbursements	**2. Geographic disparities and accessibility issues** • Disparities between Western and Eastern Slovak regions • Obligation to travel in order to get appropriate treatment • Acceptance of the obligation to travel for treatment as long as it is in Bratislava or other western healthcare facilities
**3. Decision-making and trust in the healthcare provider** • Desire for continuity of care • Involvement of cancer patients in decision-making processes	**3. Psychological burden, support and alternative coping mechanisms** • The emotional burden of cancer • Communication barriers with healthcare providers • Social support, faith and religion
**4. Malfunctioning and corruption in the healthcare system** • Lack of sufficient resources and time • Excessively long waiting lists • Corruption and systemic dysfunctionality	**4. Decision-making challenges and relationship with healthcare provider** • Communication gaps and incomplete information • Necessity of trust in the healthcare provider to participate in decision-making • Unequal opportunities of participation in decision-making
**5. Lack of information and use of the Internet** • Lack of information provided by healthcare providers and institutions • Absence of preventive measures • Lack of information on the aftermath and post-treatment care • Divergent opinions on the use of Internet	**5. Lack of information and use of the Internet** • Desire of independence, but barriers to access to necessary information • Concern about the reliability and accuracy of online information

### 3.3 Themes emerged in Croatia

Transcripts of discussions with Croatian patients resulted in 6 main themes that are described below, together with sub-themes and example quotes.

#### 3.3.1 The emotional impact of a cancer diagnosis and coping strategies

Croatian patients’ different emotional triggers and responses, together with coping strategies, are described. The first sub-theme comprises the heavy emotional burden that Croatian patients experienced because of the nature of the oncological diagnosis and the difficult treatment paths and their side effects. Patients reported having feelings of fear, sadness, and uncertainty about their future. These negative emotions made the treatment less tolerable and daily life difficult to manage, especially in patients with children; more than one patient reported having had a divorce as a consequence of the change in the relationship dynamics after the cancer diagnosis. Below, an example quote from the transcripts, that is emblematic of this sub-theme, is reported.

*“We are never healthy because we don’t know when it will come back so we fall into depression … with the recurrence … it’s not easy to do it all over again … a lot of women get divorced because their partners reject them.”*

Another sub-theme emerged concerning the lack of empathy from healthcare providers. Croatian patients often reported a complete detachment of their providers, accompanied by feelings of abandonment and confusion. Patients talked about feeling neglected or dismissed by healthcare providers, sometimes leading to frustration and isolation. This is displayed in the following quote extracted from the transcripts:

*“I called the doctor and he said I was talking nonsense and hung up on me … that hurt me the most … the attitude of my doctor … she never once came to see me.”*

A third sub-theme, strictly connected with the previous ones, emerged from the transcripts. Patients reported unequal access to psycho-oncological support in the Croatian healthcare system as well as the stigma surrounding mental health issues, not only in the context of cancer, but in the general population too. Patients seemed embarrassed or totally avoided the topic of psycho-oncological support, only a small number of patients admitted that they needed support or they had accessed it. In addition, according to patients, healthcare providers seemed to evade the topic of psycho-oncological support, or psychological aspects, as well. Two example quotes are provided below.

*“I didn’t feel the need for psychological support at all, I would solve it myself in my room … I would cry a little then go outside”.*

*“We all need psychological help, but I didn’t get it. I’m quite independent and hardworking, so that’s how I solve all these problems. But in general, nobody told me anything about it.”*

The final sub-theme regards the alternative coping mechanisms and strategies that patients adopted to face the emotional distress or the hardship that came with the cancer diagnosis and treatment when they did not want psycho-oncological support or did not have the opportunity to receive it. Patients highlighted the importance of social support from family and friends as well as physical activity and faith in their religion. Below, a quote from the transcripts indicating the crucial role of family members for emotional support.

*“I didn’t ask for a psychologist, I don’t need it, I found a way to deal with it … I had a close cousin and talking to him helped me a lot.”*

#### 3.3.2 Socioeconomic impact

This theme emerged consistently among Croatian cancer patients who reported facing economic challenges, such as direct and indirect costs of their cancer diagnosis. Firstly, patients expressed their impossibility of getting part-time contracts or sick leave benefits due to their illness. This led, for the majority of them, to indirect costs as losing a part of their income, if not the entirety, and direct non-medical costs as having difficulties in affording travel costs for treatment or exams, as well as basic necessities. This situation caused the patients to resort to detrimental financial coping behaviours as working even if they had to rest or were too sick to sit at a desk or go to the office.

A connected sub-theme is that of the income loss because of the total inability to work for a long period of time. Patients experienced financial strains, but could not work at all because of their serious condition or physical impairment. An example quote extracted from the transcripts is provided below.

*“I was unable to work, I was missing a lot from work, my boss told me that I should at least come and sit because there were conflicts as the one key person was missing … but I really couldn’t so I got fired.”*

Lastly, a third sub-theme, regarding societal and contextual risk factors, emerged with regards to the lack of awareness about financial support and reimbursements for medical expenses and travel costs. Patients complained about not being adequately, or at all, informed about the available resources and subsidies allocated to alleviate financial burdens associated with cancer and cancer treatment. The majority of patients were unaware of any type of financial support and all the patients reported that this kind of information is never provided by healthcare professionals or general practitioners. The small number of patients that knew about these resources did learn it by chance in the waiting rooms, reading newspapers or talking to friends and family. An example quote from the transcripts about the lack of information about financial support is provided below.

*“It’s scandalous that the patient does not receive the information after leaving the hospital … because my finances and my family’s finances were obviously affected and there is no information provided about financial assistance.”*

#### 3.3.3 Decision-making and trust in the healthcare provider

The relationship with healthcare providers and its implications for active participation in decision-making processes were vastly discussed among Croatian patients. The first emerged sub-theme was that of the desire for continuity of care reflecting patients’ preference to have just one oncologist to refer to and establish a long-term medical relationship with. Indeed, several patients expressed a desire to choose their healthcare provider and maintain consistency in their medical team throughout their treatment journey, as well as in the follow-up phases, fostering a sense of trust and familiarity. Below, an example quote from the transcripts is provided.

*“What is missing is having your own internist oncologist to refer to … because when you enter the system they just send you around like on an assembly line, you cannot choose anything.”*

A second sub-theme, closely related to the first one, regards the involvement of cancer patients in decision-making processes. Croatian patients underline the importance and their need of trust and collaboration in and with the healthcare providers, especially when it comes to making decisions regarding treatment or surgery. The majority of patients positively valued being involved in decision-making processes and appreciated healthcare providers who prioritised communication, transparency, and patient autonomy. The need for trust was highlighted by all the patients, indeed, they reported it as the main requirement in the relationship with healthcare providers. In particular, some said that they asked to change their provider because they did not trust him and, thus, could not make any decision or undergo any treatment or surgery. Finally, some patients expressed their preference of not being involved in the decisions, as they would rather let the healthcare providers decide or advise; clearly, for Croatian patients delegating the decision to the provider entailed a relationship built on trust. An example quote on the dynamics of the patient-provider relationship is provided below.

*“They offered an option for me to decide, in my opinion it’s a matter of trust. I decided to trust them and accept what they suggested.”*

#### 3.3.4 Malfunctioning and corruption in the healthcare system

During the focus group discussions, patients outlined several problems and malfunctioning in the Croatian public healthcare system. The first unfolded sub-theme concerns the lack of sufficient resources and time constraints highlighting the consequent challenges for patients and healthcare professionals. Croatian patients expressed frustration when systemic issues prevented the timely and appropriate delivery of efficient and comprehensive care. The majority of patients complained about the insignificant amount of time the doctors can make for them because medical personnel are lacking as well as dedicated rooms. These aspects are closely connected to the second sub-theme regarding excessively long waiting lists. Indeed, as there are insufficient healthcare providers for the great number of patients in need of medical attention, it takes a prolonged period of time for patients to book exams, consultations, tests or even receive a diagnosis and start treatment. The extended waiting times cause anxiety and emotional distress to the patients, as well as a worsening of symptoms. Below, a quote from a patient who waited nearly a month to obtain a certified diagnosis.

*“There are long waits, this and that, we all know there are problems with people and resources … it’s really exhausting, I was so scared … I wanted to know right away”.*

The last sub-theme regarding the malfunctioning of the Croatian public healthcare system exposes a distressing reality characterised by pervasive corruption and systemic dysfunctionality. As a matter of fact, Croatian patients reported experiences, direct of others, of encountering unethical behaviour, instances of corruption for the financial gain of providers or institutions, and administrative disorganisation that compromised the quality of care and the patients’ trust in the public healthcare system. Croatian patients also shared they succeeded in obtaining an appointment or being tested within a few days because they knew a nurse or a doctor working in the centre they intended to go to. In addition, bureaucratic obstacles and administrative unnecessary complications further increased patients’ frustration and confusion. Two example quotes are provided below.

*“It was just a matter of the code of the referral, but later I found out that the more patients the general practitioner sends to the lab, the higher the commission they get … so then I changed the doctor.”*

*“If you don’t complain nobody listens to you, I had a disability, but I had to fight and go through several bureaucratic procedures to finally obtain a certificate and the help I needed.”*

#### 3.3.5 Lack of information and use of the internet

Another theme emerged from focus group discussions with Croatian patients and it entails the challenges faced by cancer patients in accessing comprehensive and timely information, underlining the crucial role of healthcare providers and official institutions in providing adequate information or reliable online sources to empower patients in making informed decisions about their care. A critical sub-theme, highlighted by Croatian patients, is the lack of information provided by healthcare providers and institutions. This sub-theme concerns the underlying necessity for patients to receive comprehensive and accurate information. Croatian patients expressed their frustration and the barriers they had to face while seeking guidance and clarity over their diagnosis, treatment options, available support services, social benefits, and, most importantly, their rights as cancer patients. A quote on this topic is provided below.

*“It would have been easier to get help and information from the doctor for the centre for social welfare and things like that … because what I know I heard in the waiting rooms like the right to additional healthcare insurance which is very important.”*

The second sub-theme regards the absence of preventive measures; patients complain about the lack of information regarding prevention, particularly for younger generations, and call for increased efforts in this regard. Additionally, they mentioned the absence of screening programs, proactive initiatives aimed at early detection and prevention in schools, workplaces and television or online advertisements. One participant phrased the issue as follows:

*“Everybody is talking about prevention, but there are no preventive measures for young women … what are we talking about? there is no prevention for women under 40…much work should be done in this direction”.*

Patients also reported experiencing a lack of information on the aftermath (third sub-theme). In fact, they were concerned about the absence of information regarding post-treatment care and the long-term effects of cancer treatment. In addition, Croatian patients highlighted the importance of being informed about nutritional guidelines, exercise recommendations, and follow-up procedures to support their recovery and enhance their quality of life. One example quote is reported below.

*“That part is missing … how to get back to normal after the treatment is over … regarding the immune system, exercise, nutrition…”*

While talking about the lack of accessibility to information, Croatian patients voiced their opinions on the use of the internet (fourth sub-theme) and online sources to search for information regarding their medical condition. While relying on internet resources to expand the information received from healthcare providers, patients also seek reliable sources such as clinical research articles and official websites. However, Croatian patients complained about the abundance of misinformation online; they do not know if the information they find can be trusted. Thus, patients would want to receive suggestions on reliable sources in order to be able to gain useful information on their own instead of relying on the healthcare providers who, on their side, do not appear to have time or interest in sharing crucial facts or recommendations with them. Still, some patients prefer talking to their healthcare professional instead of searching online, both because they prefer to have a relationship with the provider and because they do not know how to navigate the internet. One of the participants stated:

*“I would prefer the doctor to give all the information, but I’m aware that in the current conditions it’s very difficult due to the number of patients. And there is a lot of incorrect information on the internet. But it would be great if there was a reliable platform.”*

#### 3.3.6 Expressed needs and suggestions

The last theme reflects the expressed needs and suggestions of Croatian cancer patients, highlighting areas where improvements in communication, organisation of care, accessibility, and continuity of care could enhance their overall experience, survival and quality of life. As it is qualitatively different from the other themes, all the suggestions, as well as example quotes, are separately reported in **Table 4**.

**Table 4:** Suggestions from Croatian Patients.

Suggestions	Quotes
Provide clearer information dissemination regarding appointment scheduling, receiving test results, and available support services via text message or email.	*“It would be also useful to get text messages to know where to go for an appointment and when so you don’t have to wait in crowds.”*
Establish centralised cancer care centres that would serve as comprehensive hubs where patients can access all necessary services in one location and avoid travelling between different facilities.	*“Everything should be in one place, and should have the opportunity to get treatment and exams all in the same centre, because otherwise is a true struggle.”*
Assign to each patient a dedicated healthcare professional to provide personalised care and guidance ensuring easier transitions between the various stages of care.	*“It would be good if each patient had a single person accompany them throughout the entire process.”*
Implement better signalling or guidance systems within healthcare facilities.	*“We need better signalling, a coordinator or a nurse that can direct the patient in the centres.”*

Several suggestions emerged in response to the needs and barriers that have been described in the previous themes. In order to improve communication channels, patients advocate for clearer information dissemination regarding appointment scheduling, receiving test results, and available support services. The patients’ proposal would be receiving text message reminders for scheduled appointments and having test results sent via email, in order to reduce uncertainties and have a better organised healthcare system.

In addition, patients underlined the logistical complications associated with navigating multiple healthcare facilities for tests and treatment. Their suggestions would be to establish centralised care centres that would serve as comprehensive hubs where patients can access all necessary services in one location avoiding or reducing the burden of travelling between different facilities.

A third point for improvement, suggested by Croatian patients, would be assigning to each patient a dedicated healthcare professional to provide personalised care and guidance ensuring easier transitions between the various stages of care. In the patients’ opinion, this solution would address their strong preference for being followed by a single doctor throughout their cancer journey.

Lastly, to improve accessibility and comfort in obtaining healthcare services, Croatian patients suggested the implementation of better signalling or guidance systems within healthcare facilities.

Collectively, these suggestions display the patients’ unmet needs regarding a more patient-centred and efficient healthcare system that prioritises their needs and promotes better outcomes.

### 3.4 Themes emerged in Slovakia

Transcripts of discussions with Slovak patients resulted in 5 main themes that are described below together with sub-themes and example quotes.

#### 3.4.1 The cancer taboo

This first theme, emerged from Slovak patients, underlines the urgent need to destigmatise cancer in Slovak society in order to enhance access to reliable information and support networks. Promoting open dialogue to empower patients, as well as the general population, in adopting prevention measures, adhering to screening programs, and finding the best comprehensive care would be one of the goals to achieve in Slovak patients’ opinion.

The first sub-theme, indeed, regards the extensive taboo associated with cancer reported by Slovak patients; they complain it is never spoken of, contributing to significant challenges in accessing information and support. Patients also expressed frustration over the lack of open dialogues about cancer prevention, diagnosis, and treatment. An example quote is provided below.

*“We don’t know anything Doctor, we don’t know anything about prevention or what a cancer diagnosis even is, nobody talks about it! Like it is very rarely spoken about!”*

The following sub-theme emerged consequently. Slovak patients, in fact, complained about the absence of active online communities or Facebook groups specifically tailored to Slovak cancer patients. Instead, they came across these support groups and communities from other countries such as France, the United States of America, and the United Kingdom. Slovak patients expressed feelings of isolation and barriers to accessing relevant information regarding their rights, social benefits and financial assistance, usually country-specific. A cancer patient reported her experience as follows:

*“I found some Facebook and Instagram groups, but abroad! You don’t talk about cancer here, most people completely avoid it!”*

#### 3.4.2 Geographic disparities and accessibility issues

Slovak patients reported that access to quality cancer care in Slovakia is impaired by significant geographic disparities and consequent challenges in accessibility. Three main sub-themes emerged from the focus group discussions regarding geographic disparities.

Initially, Slovak patients highlighted existing disparities between the West and the East of Slovakia, reporting that the best comprehensive cancer care can be received only in Bratislava and maybe a few other cities in the Western regions. Patients complained about relevant differences in healthcare infrastructure, resources, and providers’ expertise resulting in pervasive disparities in the access to specialised and innovative treatments and experienced medical professionals. Below, a representative statement regarding this issue.

*“The healthcare system in Slovakia, we know it’s not the best, especially in the East and in small towns … it is like in the East they are in another country.”*

As a consequence to these geographic disparities, a second sub-theme emerged regarding the obligation to travel in order to get appropriate treatment, resulting in additional direct, but non-medical, costs. Slovak patients underlined the financial, as well as physical, strain associated with frequent travel for cancer treatment, tests or exams in addition to accommodation expenses, and transportation and medical costs. Moreover, patients expressed their frustration with regards to individual household and contextual risk factors, such as the lack of insurance coverage for certain procedures and treatments, particularly mastectomy, regardless of positive genetic testing. These factors, reportedly, had a psychological impact on the patients, resulting in psychological financial responses, as negative feelings towards financial experiences, manifesting in the form of financial rumination. One patient described his/her experience as follows:

*“I had to deal with many travels, but the ambulance came to pick me up … I also had to move to Prague, so to another country, to go to a specialised centre where I underwent radiotherapy, obviously I had to pay for it myself.”*

Nevertheless, despite the challenges associated with travel, a third sub-theme emerged regarding the patients’ preference for travelling to get treatment as long as they are being treated in Bratislava or other western healthcare facilities. The patients would rather endure the economic, energetic, and time costs than risk being treated in eastern hospitals that are inadequate. As a matter of fact, some patients reported knowing other cancer patients who had to be treated in the East, because they couldn’t afford or manage to travel, and have died. Two relevant quotes are reported.

*“I had to move because I knew that if I stayed to be treated near home it wouldn’t end well, I know people who were treated there and it didn’t go well…”*.

*“For me, the heaviest thing, especially psychologically, is when after therapy I was transferred from NOU (Bratislava) for check-ups, and those I had to do in a hospital near my home in the East … it was a blow because I felt good at NOU and I trusted it, so I felt really abandoned, dumped, and I didn’t trust it here.”*

#### 3.4.3 Psychological burden, support and alternative coping mechanisms

The third theme emerged from the discussions with Slovak patients concerns their challenges and preferences regarding psychological support and alternative coping mechanisms.

The first sub-theme regards the emotional burden of cancer. Patients reported significant emotional suffering due to the cancer diagnosis or treatment describing feelings of fear, vulnerability and psychological distress. Some patients expressed the need to have had the opportunity to receive psycho-oncological support to ease the emotional burden. A quote reporting such need is the following:

*“During chemotherapy, the person is very weak, and this also greatly affects the psychological aspect. I would have really appreciated it if psychological support had been offered to me, I really needed it.”*

Further, patients expressed their frustration encountering communication barriers with healthcare providers who often avoid discussing any patients’ concerns that are not strictly medical (second sub-theme). In addition to the limited time the providers can afford to dedicate to them, Slovak patients perceived their lack of interest in addressing psychological aspects or emotional distress. The absence of communication and information provision regarding mental health and the availability of psychological support leads to barriers and disparities in the access and use of such support, exacerbating feelings of isolation and additional distress. One participant referred to such absence of information as follows:

*“I didn’t know you could have psychological support, they don’t tell you anything about it … I found out through these focus groups actually … I would have needed it.”*

The final emerged sub-theme regards the alternative coping mechanisms that patients enacted or resorted to in order to ease their emotional distress and find some support in facing the many challenges of cancer diagnosis and treatment. Patients reported that strategies such as seeking support from family members or friends, relying on religious faith, and connecting with other patients online, or within the healthcare facilities, helped them to deal with all the emotional struggles they have been through and to be more resilient. Some patients highlighted that the possibility of attending church services and their faith in God was the most important support for them and their families, more than any other type of support. An example quote is provided below.

*“I am very religious, so my faith in God really helped me to overcome everything, even psychologically.”*

#### 3.4.4 Decision-making challenges and relationship with healthcare provider

The involvement in the complex dynamics of decision-making processes and its connection with the relationship between patients and healthcare providers emerged as a whole separate theme among Slovak patients.

The first sub-theme concerns communication gaps and incomplete information. Slovak patients express their preoccupation with the incomplete provision of information regarding treatment options, risks, and other medical details. Many patients reported instances where they were not sufficiently informed about potential side effects or alternative and less intrusive treatments impacting their ability to make informed decisions. For example, during the focus group discussions, more than one patient complained about the fact that they were not informed about the possibility of freezing the eggs before therapy, or even that the therapy might impact their fertility, and, thus, after the treatment they were no longer able to have children as they wished. Two participants stated as follows:

*“I had to insist on having proton therapy because I am very young, and in the end, after many insistences, I was able to do that and not the classical radiotherapy.”*

*“To me, this was not communicated, and now that my husband and I were thinking about it, we unfortunately found out that it is no longer possible, but we were not even offered to freeze the eggs.”*

The second sub-theme concerns the patients’ necessity to trust and confide in the healthcare providers in order to seek their guidance and go through shared decision-making processes. Indeed, the majority of Slovak patients expressed their need to have a deeper relationship with their oncologist in order to ask them for advice and trust that what they are suggesting might be the best option for them. Patients report the need to rely on medical expertise as well as advocate for their involvement in decision-making processes. For example, one of the participants stated:

*“I think that the patient should not have complete decision-making freedom because then there is a risk that the patient will not seek treatment and will not survive … unfortunately, I have known people like that.”*

Lastly, another sub-theme emerged, regarding unequal opportunities and experiences with healthcare providers with regards to shared decision-making processes. Unfortunately, while some patients did have the opportunity to participate in decisions and listen to healthcare providers’ opinions, others felt isolated and disempowered with little to no possibility to interfere with decisions, ask for suggestions, or even be consulted. However, despite the majority of patients reporting the benefits of their involvement in the treatment decisions, some patients expressed they preferred the healthcare provider making all the decisions without interfering as they knew the doctors had a degree and thus were capable and aware of what they were doing. Nonetheless, other patients argue that healthcare providers do not possess the necessary time and energy to make personalised decisions for each patient, so it is right and necessary to be informed of all the possibilities and consequences. Here are two example quotes about this theme.

*“I have never decided anything, I have never asked if there were alternatives … I am from an older generation, we do not involve ourselves in these matters, if a doctor tells me something, then that’s it.”*

*“I had the feeling of not having had a say in what would happen; the doctor said, ‘we’ll do chemotherapy, and we’ll see how to proceed,’ and I didn’t decide anything.”*

#### 3.4.5 Lack of information and use of internet

Finally, a last theme emerged concerning the Slovak patients’ use of the internet to search for information regarding social or financial support, bureaucratic steps and policies, medical advice, and cancer patients’ rights, in response to a lack of information from healthcare providers, general practitioners and healthcare institutions.

Firstly, patients expressed a strong desire to take charge and be independent in managing most of the aspects of their disease, aside from the medical ones, but faced barriers in accessing sufficient information from healthcare providers, official institutions and family doctors (first sub-theme). The latter issue led the majority of the patients to search the internet, utilising search engines and online communities to gather insights about financial aids, disability benefits, nutrition, physical therapy, and social support. Patients reported a lack of awareness and proactive interest from healthcare providers regarding collateral issues acting as a barrier to useful and necessary information gathering. As a patient expressed:

*“Two of my friends developed lymphedema … because there was no possibility of having a physiotherapist, we didn’t know what to do with this arm … who to ask, where to go … so I asked other patients in a Facebook community from France.”*

Further, despite patients’ concern about the reliability and accuracy of online information, they reported turning to internet sources as a primary means of information (second sub-theme). However, Slovak patients acknowledge the limitations of online resources but find them to be a valuable alternative or supplement. While the majority of patients clearly stated their preference for searching for information online, still a number of patients reported trusting the healthcare provider only and did not feel like needing additional information. Indeed, this group of patients believed that if healthcare providers did not provide other information, it was either unnecessary or not required. Overall, the majority of patients reported that they mostly received information regarding social assistance, financial aids and other types of support, from online groups or sources. Two excerpts from the interviews are reported below.

*“For information about therapy, the illness, and disability, a non-profit organisation that I found on the internet helped me a lot, and in general, I found and searched for a lot of information on the internet … I think unfortunately doctors don’t have time to explain to us because they are overwhelmed.”*

*“Let’s say that I have always preferred consulting my doctor, who has always listened to me and advised me … He has a medical degree after all and knows what is necessary to know and what is not.”*

## 4 Discussion

The present study investigated possible disparities in access to cancer care within Central and Eastern Europe, narrowing attention to the voices and experiences of cancer patients from Croatia and Slovakia. Through online focus group discussions, participants shared their insights and perspectives highlighting various barriers and unmet needs encountered during, or after, their cancer journeys.

Distinct themes emerged from these discussions, reflecting the unique contexts of Croatian and Slovak patients, yet revealing extensive patterns that emphasised shared challenges across both groups. These converging themes provided complementary perspectives on the multifaceted nature of cancer care disparities in CEE countries.

While common barriers such as the lack of information from healthcare providers, the use of the internet, and the emotional burden of cancer were observed, country-specific disparities and barriers shed light on the intricacies of each nation’s healthcare landscape highlighting differences between the Slovak and Croatian healthcare systems. Similarities and differences in the themes from both countries are described below.

Among the various experiences and testimonies provided by both groups of patients, that of the emotional burden of cancer appears to be similarly prevalent. Both Croatian and Slovak cancer patients reveal a shared struggle with the psychological burden that came with their diagnosis, particularly with the fact that it was not addressed properly or at all. In fact, both groups seek comfort in alternative coping mechanisms, such as social support and religious faith. These findings are coherent with previous literature highlighting the universal challenges with mental health stigma in Central-Eastern Europe (17). While these nations have looked to Western models for inspiration, challenges persist, as evidenced by the triple barrier identified by Muijen and McCulloch (18): stigma, resource limitations, and workforce shortages. In the context of Croatia and Slovakia, patients facing cancer-related psychological burdens encounter similar obstacles, including the limitation of resources and shortages of medical personnel. Possibly, such barriers prevent patients, healthcare providers, and institutions from recognising the importance of the psychological wellbeing of cancer patients in Slovakia and Croatia. Nonetheless, the existing literature underscores the importance of integrating psycho-oncological support into cancer care frameworks, which not only enhances quality of life, but also increases survival rates among cancer patients (19). Further research should focus on developing culturally sensitive mental health programs and policy advocacy for increased resource allocation, ensuring that the psychological needs of cancer patients are adequately met (20).

Another similarity between the experiences of Slovak and Croatian cancer patients regards patients’ involvement in shared decision-making processes. Both groups emphasised the significance of trust in their relationship with healthcare providers, valuing communication, transparency, and patient autonomy. However, challenges such as communication gaps and incomplete information were reported by both groups of patients, leading to concerns about their ability to make informed decisions. Despite these challenges, patients from both countries expressed their preference for being involved in treatment decisions, highlighting the importance of trust and collaboration with healthcare providers in navigating their cancer care journey. These findings align with previous research indicating variations in patients’ preferences for involvement in decision-making processes. In addition, findings suggest that the more patients confide in the healthcare providers the more they want to be involved in decision-making; however, gender differences are to be considered (21). The literature underscores the value of patient-centred care and the need for healthcare systems to foster communication, transparency and collaboration. Addressing this issue could enhance patient autonomy and satisfaction, ultimately leading to better treatment outcomes and compliance, lower mortality rates, better quality of life and a lighter work burden for healthcare providers (22).

Lastly, both Slovak and Croatian patients expressed their frustration with a substantial lack of information from healthcare providers and institutions regarding medical aspects as well as necessary health and social support needs such as nutrition and financial aid. Despite recognising the limitations of online information concerning its reliability, many patients still viewed the internet as a valuable resource. Both groups emphasised the importance of improved communication and information dissemination within the healthcare system, especially regarding reliable internet sources or information. Similar findings can be found in previous research exploring barriers and facilitators of cancer-related online information-seeking behaviours and reasons for disparities in cancer patients from Italy (13, 23). Improving the provision of accurate and comprehensive information is crucial for patient empowerment, enabling better health outcomes and longer survival. By enhancing communication strategies and ensuring the availability of reliable information, healthcare systems can better support cancer patients in navigating their treatment and accessing necessary resources (24).

As for the differences between the themes emerging from Croatian and Slovak patients, a quite interesting result is that of the theme of socioeconomic impact, which emerged prevalently from the discussions with Croatian patients. Indeed, they consistently highlighted various indirect costs stemming from their cancer diagnosis, including difficulties in securing part-time contracts or sick leave benefits, leading to income loss and struggles in affording direct medical costs and basic necessities. Unfortunately, these costs, for some households unbearable, often can lead patients to adopt dangerous financial coping behaviours in order to increase liquidity and resources. Indeed, as reported by the Organisation of European Cancer Institutes (OECI) Task Force in the recommendations on a conceptual framework for the SEI of cancer, patients’ can make dysfunctional adjustments such as limiting or forgoing treatment that can have a heavy impact on their survival and general quality of life (25). However, despite it being a prominently discussed theme among Croatian patients, it is almost total absence in Slovak patients does not appear to align with the financial burden of cancer as highlighted in previous literature across most CEE countries such as Bulgaria and, note, Slovakia (26). Indeed, CEE countries allocate substantially less funding for cancer care than other EU countries. On average, CEE countries spend 2.5 times less than Western European countries on oncology drugs, financial aids, education and preventive measures per new cancer case. These findings indicate the urgent need for patients, healthcare providers, researchers and policymakers in CEE to advocate for greater financial support and evidence-based resource allocation to improve cancer care outcomes in the regions (27). This discrepancy may be linked to the small number of Slovak patients participating in the current study, as well as their geographic location (mostly Bratislava region and western-central Slovakia). As a matter of fact, Slovakia exhibits significant regional income inequality, ranking among the highest in Europe, with a sharp contrast between the GDP (gross domestic product) per capita in Bratislava, which significantly surpasses the rest of the country, and the Eastern region, characterised by its high poverty rate and numerous disadvantaged households (28).

Coherently with the aforementioned literature, geographical disparities in Slovakia emerged as a pervasive issue from the discussions with Slovak patients. Indeed, the income inequality among Slovak regions adds to, and generates, disparities in the quality and accessibility of cancer care, health infrastructure, and expertise, as well as education and literacy of the population (29, 30). Furthermore, it is worth noting that the prevalence of poverty, low education levels, and limited health literacy in Slovakia contribute to cancer being viewed as a taboo topic in the majority of regions outside of Bratislava impacting on cancer awareness and consequently increasing mortality rates and shorter survivals (31). This sentiment was particularly evident in the focus group discussions with Slovak patients, who lamented the lack of information, patient associations and online communities as a direct result of the taboo surrounding cancer in Slovakia, highlighting the profound impact of socioeconomic factors on perceptions and attitudes toward cancer care in the country (32). Addressing these disparities requires a multifaceted approach, including infrastructure improvement, equitable resource distribution and education initiatives to raise cancer awareness in underserved regions and among the aforementioned population strata.

Mentions of geographic disparities also emerged from Croatian patients in relation to a specific theme that arose during the focus group discussions. As a matter of fact, Croatian patients reported experiences of malfunctioning and delays in the healthcare system, especially in smaller cities or rural areas. In addition, Croatian patients shared concerns and experiences regarding episodes of corruption or unfairness on the part of the healthcare providers. This is consistent with previous research, especially with a study conducted by European Commission experts, which concluded that corruption in medical service delivery remains one of the main challenges especially in Croatia and in many Eastern and Southern European Member States (33). Addressing these geographic and systemic disparities is crucial for improving cancer care in these regions. Strategies such as increasing transparency, implementing robust anti-corruption measures, and investing in healthcare infrastructure in rural areas could mitigate these issues.

Finally, the last observed difference between Slovak and Croatian patients’ discussions lies in the emerged suggestions provided exclusively by Croatian patients. These suggestions illuminate additional systemic challenges within healthcare systems and underscore the necessity of considering patient perspectives for comprehensive reforms. Highlighted areas for improvement include communication, care organisation, accessibility, and continuity of care. Suggestions such as clearer information dissemination, establishment of centralised care centres, assignment of dedicated healthcare professionals, and implementation of better signalling or guidance systems within healthcare facilities exemplify the need for systemic enhancements. These results, reported in **Table 4**, could represent useful implications for further research. Indeed, integrating patient needs and perspectives is essential for addressing these challenges and advancing towards a more patient-centred healthcare approach. One noteworthy suggestion from Croatian patients, regarding the implementation of text messages for appointment reminders and test result notifications, underscores the potential for cross-country learning and adaptation of successful practices to address systemic challenges in healthcare delivery. Interestingly, similar systems are already in place in Italy and other Northern-Western European countries, such as Estonia, the Netherlands, Denmark and Sweden, suggesting the feasibility and potential effectiveness of adopting this practice to improve communication and patient engagement across healthcare systems (34). By prioritising patient needs and the perspectives emerged, healthcare systems could take significant strides towards fostering a more patient-centred approach to healthcare delivery, ultimately leading to improved outcomes and experiences for all stakeholders involved. In addition, further research addressing comprehensive cancer care in CEE countries is needed, as well as official guidelines for cancer care and awareness campaigns, in order to decrease the existing cancer disparities between Northern-Western and Central-Eastern Europe (35). Lastly, attentive focus should be placed on mental health, advocating for strategies such as post-graduate training, regional collaboration, and capacity building in research management. By elevating the discourse on mental health in CEE countries, the agenda of the global psycho-oncological field can be advanced and equitable access to quality cancer care ensured (36).

Despite the valuable insights gained from the current study, it is important to acknowledge its limitations. Firstly, the sample size of both Croatian and Slovak patients may limit the generalisability of the findings to broader populations within CEE countries. Moreover, both samples were susceptible to bias as Croatian patients were all treated in Zagreb, while the majority of Slovak patients resided in the more advantaged regions of the country. Additionally, a critical limitation to consider is that the Slovak patients were almost entirely treated within private healthcare settings. This context may have introduced a bias in their reported experiences, particularly regarding SEI. Patients in private healthcare often encounter different financial dynamics compared to those in public healthcare systems, potentially leading to an underrepresentation of the financial constraints commonly experienced by the broader Slovak cancer patient population. This factor must be considered when interpreting the findings, as the financial experiences of these Slovak patients might not fully reflect the challenges faced by the wider community. It is pertinent to acknowledge the encountered challenges in recruitment, possibly stemming from societal taboos surrounding cancer and a concomitant lack in awareness within the Slovak population. Additionally, the qualitative nature of the research inherently introduces the potential for subjectivity and bias in the interpretation of participant responses. Furthermore, the study focused solely on patients’ perspectives, without incorporating perspectives from healthcare providers or policymakers, which could provide valuable insights into systemic challenges and potential solutions (13).

However, despite these limitations, the study offers several strengths. The inclusion of both Croatian and Slovak patients provides a comparative analysis that enriches our understanding of the nuances in cancer care experiences across different contexts, often remaining unexplored. Moreover, the qualitative approach allowed for in-depth exploration of patient perspectives, uncovering rich insights that may inform future research and interventions aimed at improving healthcare delivery for cancer patients in Central and Eastern Europe. Lastly, the use of the SRQR guidelines and the SEI conceptual framework to conduct and correctly report the methodology and the results of the current study could represent an additional strength.

## 5 Conclusion

In conclusion, this study highlights the importance of integrating patient perspectives to address systemic challenges in cancer care delivery in CEE countries. The insights gained, particularly from Croatian patients’ feedback regarding the implementation of text message reminders, underscore the potential for cross-country learning and adaptation of successful practices. Moving forward, there is a critical need for comprehensive cancer care initiatives, official guidelines, and increased attention to mental health advocacy in the region. To translate these findings into actionable improvements, several steps, such as enhancing communication systems, developing comprehensive cancer care guidelines, increasing mental health support and awareness and addressing financial barriers are recommended. While acknowledging the limitations of this study, including sample size constraints and the qualitative nature of the research, its comparative analysis and adherence to methodological guidelines provide valuable insights that can inform future research and interventions. Addressing these limitations through broader and more inclusive research will be crucial for developing a comprehensive understanding of cancer care disparities and implementing effective solutions in CEE countries.

## Data Availability

The datasets used and analysed during the current study are available from the corresponding author on reasonable request.
