# Supplementary material for: Patients’ perspectives on cancer care disparities in Central and Eastern European countries: experiencing taboos, misinformation and barriers in the healthcare system
Source: Front Oncol. 2024 Aug 9;14:1420178. doi: 10.3389/fonc.2024.1420178 (PMC11341380; doi:10.3389/fonc.2024.1420178)
Supplement: Supplementary file 2 [file Table_2.docx]

**Recruitment Process**

**Participants’ selection criteria**

**Exclusion**

**Inclusion**

- Non-residents of Croatia or Slovakia.
- Younger than 18 years.
- No internet access.
- No cancer diagnosis or not caregivers to cancer patients.
- Age: 18 years or older.
- Diagnosis: cancer patients, survivors or caregivers.
- Residence: Croatia or Slovakia.
- Language: fluent in Croatian or Slovak.
- Technology: efficient internet connection and technological devices.

5 Focus Group sessions

N=15 (all treated within the public healthcare system)

3 Focus Group sessions

N=11 (4 treated within the public healthcare system, 7 in private centres)

Recruited in: hospitals, oncology centres

Recruited through: social media, hospital websites, cancer patients' organizations

**Croatia**

**Slovakia**

**Participants**
